# Resolved hyperthyroidism before IVF is not associated with improved cumulative live birth rates: a retrospective cohort study

**DOI:** 10.3389/fendo.2026.1749355

**Published:** 2026-06-02

**Authors:** Huizi Jin, Jiejun Luo, Menglu Wu, Yuxuan Fang, Zijin Xu, Lin Zhu, Mingzhu Cao, Zhu Liang, Jing Wang, Yanshan Lin, Yixuan Wu, Wenju Peng, Haiying Liu, Jian-Qiao Liu

**Affiliations:** 1 Department of Obstetrics and Gynecology, Center for Reproductive Medicine; Guangdong Provincial Key Laboratory of Major Obstetrics Disease; Guangdong Provincial Clinical Research Center for Obstetrics and Gynecology; Guangdong-Hong Kong-Macao Greater Bay Area Higher Education Joint Laboratory of Maternal-Fetal Medicine; The Third Affiliated Hospital, Guangzhou Medical University, Guangzhou, China; 2 Department of Biomedical Engineering, City University of Hong Kong, Hong Kong, Hong Kong SAR, China; 3 Division of Gynecologic Oncology in the Department of Obstetrics and Gynecology, The First Affiliated Hospital of USTC, Division of Life Sciences and Medicine, University of Science and Technology of China, Hefei, China; 4 Department of Gynecologic Oncology, Anhui Provincial Cancer Hospital, Hefei, China

**Keywords:** cumulative live birth rate, fertility, hyperthyroidism, *in vitro* fertilization, live birth rate, resolved hyperthyroidism

## Abstract

**Background:**

Hyperthyroidism adversely impacts female reproduction, yet its association with *in vitro* fertilization (IVF) outcomes remains controversial. It remains unknown whether ovarian function recovers upon achieving euthyroidism. We compared the IVF outcomes among women with active hyperthyroidism (AH), resolved hyperthyroidism (RH), and normal control (NC) patients.

**Methods:**

This retrospective study included patients undergoing their first oocyte retrieval cycle from January 2014 to September 2022, the follow-up lasted for two years. Thyroid function (TSH, FT3, FT4) was classified using the most recent test performed within 3 months before oocyte retrieval. Patients were categorized into three groups: AH, RH, and NC group based on the thyroid hormone level and disease history. The primary outcome was cumulative live birth rate (CLBR) per aspiration cycle. Multivariable logistic regression and inverse probability weighting (IPW) were used to evaluate the association, with adjustment for baseline characteristics and treatment protocols. Subgroup analysis examined the association between hyperthyroidism duration and CLBRs in patients in the AH/RH groups.

**Results:**

17,621 patients were involved, with 257 in the AH group, 297 in the RH group, and 17,067 in the NC group. The fresh cycle LBR was significantly lower in group AH compared to NC (31.9% vs. 43.7%, P = 0.034). CLBRs were 42.3% in group AH, 44.1% in RH, and 54.9% in NC. Adjusted models revealed lower LBR and CLBR in group AH versus NC, and no significant differences between group AH and RH. In the subgroup of patients with hyperthyroidism, longer duration of hyperthyroidism was negatively associated with cumulative live birth (aOR=0.9990 (0.9985-0.9995), P< 0.001).

**Conclusions:**

Preconception hyperthyroidism was associated with poorer IVF outcomes, and longer disease duration was associated with a lower CLBR.

## Introduction

Hyperthyroidism, defined as excessive production of thyroid hormones (THs), is a critical condition with far-reaching implications for reproductive health (1, 2). Women of reproductive age are disproportionately affected, with hyperthyroidism prevalence estimated at 0.2% to 2.5% (3). This endocrine disorder, often resulting in thyrotoxicosis, disrupts multiple physiological systems, including metabolism, cardiovascular function, and the hypothalamic-pituitary-gonadal axis, all of which are essential for successful conception and pregnancy maintenance, especially among the *in vitro* fertilization (IVF) population (4, 5).

The association between hyperthyroidism and pregnancy outcomes is complex. Because THs are relevant to reproductive function, even small hormonal changes can harm the delicate balance needed for conception and pregnancy maintenance (6, 7). Understanding the impact of hyperthyroidism on pregnancy outcomes is critical for optimizing treatment protocols and improving IVF success rates. Previous studies have indicated that hyperthyroidism during pregnancy is associated with adverse obstetric outcomes, often necessitating treatment or thyroidectomy (8–11).

However, most studies focus on hyperthyroidism occurring during pregnancy, which is often transient and resolves spontaneously postpartum (12). Few studies address hyperthyroidism prior to pregnancy. For women with hyperthyroidism who desire pregnancy, achieving stable euthyroidism often requires several months or even years, during which fertility may decline, particularly in the IVF population (13, 14). Current guidelines still recommend achieving stable euthyroidism before pregnancy, especially before IVF (15, 16). An unresolved clinical question arises: Should hyperthyroidism be fully treated before attempting conception, or is it possible to pursue pregnancy during treatment? To date, there is insufficient evidence to guide this decision.

This study examines IVF outcomes in women with active and resolved overt hyperthyroidism compared with euthyroid women. The goal of this research is to determine whether IVF outcomes improved after resolution of hyperthyroidism. The findings aim to guide women with hyperthyroidism, helping them balance the need for treatment with their immediate reproductive goals.

## Materials and methods

### Study design

This retrospective study analyzed data from patients undergoing their first IVF or intracytoplasmic sperm injection (ICSI) treatments at the Third Affiliated Hospital of Guangzhou Medical University between January 2014 and September 2022. Exclusion criteria included: (1) age <20 years or >45 years, (2) use of donated or frozen oocytes, (3) transferring embryos from multiple retrieval cycles, (4) incomplete core information, including TH levels and controlled ovarian stimulation protocols, and (5) diagnosis of subclinical hypothyroidism, hypothyroidism, subclinical hyperthyroidism, thyroiditis, or thyroid cancer.

Thyroid function was classified using the most recent serum thyroid-stimulating hormone (TSH), free triiodothyronine (FT3), and free thyroxine (FT4) measurements obtained before oocyte retrieval. When multiple thyroid function tests were available, the result closest to oocyte retrieval was used. Only patients with a thyroid function test performed within 3 months before oocyte retrieval were eligible for inclusion; patients whose most recent available test was more than 3 months before were excluded. In routine clinical practice at our center, all thyroid function tests used for classification were obtained before the start of controlled ovarian stimulation (COS). Overt hyperthyroidism was defined according to our hospital’s clinical diagnostic criteria as TSH <0.30 mIU/L, FT3 >5.7 pmol/L and/or FT4 >19.01 pmol/L. the Roche electrochemiluminescence platform was used to measure these hormones, and the laboratory reference ranges were 0.30 - 4.50mIU/L for TSH, 2.63-5.7pmol/L for FT3, and 9.01-19.01 pmol/L for FT4.

Patients were categorized into three groups: (1) the active hyperthyroidism (AH) group, comprising patients with a diagnosis of overt hyperthyroidism within 3 months prior to oocyte retrieval; (2) the resolved hyperthyroidism (RH) group, consisting of patients with a previous diagnosis of overt hyperthyroidism who had discontinued all antithyroid treatment for at least 3 months before oocyte retrieval, maintained euthyroidism without medication during this period, and remained untreated throughout COS and embryo transfer; and (3) the normal control (NC) group, including patients without any thyroid dysfunction.

This study was approved by the Institutional Review Board of Guangzhou Medical University (R3-LCYJ-IIT-301-01). All patients routinely signed an informed consent for the use of their data for research purposes.

### IVF/ICSI treatment and embryo transfer

COS protocols included gonadotropin-releasing hormone (GnRH) agonists, antagonists, and individualized protocols. When at least two follicles reached a diameter of ≥18 mm, ovulation was triggered with 250 μg of recombinant human chorionic gonadotropin (hCG). Oocyte retrieval occurred 36 hours after hCG injection under transvaginal ultrasound guidance and was canceled if oocytes were expelled prematurely, patients had health issues, or for personal reasons.

For embryo assessment, cleavage embryo quality was assessed on day 3 based on blastomere number, fragmentation rate, and multinucleation. The Gardner blastocyst grading system evaluated blastocyst quality on day 5.

Available embryos not transferred during the fresh cycle were vitrified and cryopreserved using the Cryotop system for subsequent frozen embryo transfer (FET) cycles. During FET cycles, endometrial preparation protocols included programmed, natural, or stimulated cycles. No more than 2 embryos were transferred per cycle.

### Outcome measurements

Serum hCG levels and ultrasonography confirmed pregnancy. Once intrauterine pregnancy was confirmed, patients were followed up until 6 weeks postpartum via regular telephone contact. Biochemical pregnancy was defined as a positive hCG test without a gestational sac detected by ultrasound. Clinical pregnancy was defined as one or more gestational sacs observed on ultrasonography at 4 to 6 weeks post-transfer. A live birth refers to delivering one or more live infants at ≥28 weeks of gestation. Early pregnancy loss refers to miscarriage within the first 12 weeks of gestation.

The primary outcome of this study was the cumulative live birth rate (CLBR) after a single aspiration cycle. CLBR was determined by following patients for two years or until all embryos were utilized or a live birth occurred, whichever came first. At the end of the two-year follow-up, pregnancies that reached ≥14 weeks of gestation were recorded as ongoing pregnancies. For patients who did not achieve a live birth or exhaust all embryos within the two-year timeframe, a conservative assumption was made that a live birth would not occur in that cycle. Patients with canceled oocyte retrieval were excluded from cycle-level outcome analyses.

Subgroup analyses were conducted among women with a history of overt hyperthyroidism (group AH and RH). Telephone follow-up was performed to gather information on the duration of hyperthyroidism, treatment methods, perinatal, and neonatal outcomes. For AH patients, the duration was defined as the time from diagnosis to the date of oocyte retrieval. For RH patients, the duration was defined as the time from diagnosis to the normalization of TH levels.

### Statistical analysis

Continuous variables were reported as medians with interquartile ranges (IQRs) and compared using the Mann-Whitney U test or the Kruskal-Wallis test. Categorical variables were presented as frequencies with percentages and compared using the χ² test or Fisher exact test, as appropriate.

To mitigate confounding effects, three adjusted models were applied. Model 1 utilized multiple linear regression analysis for oocyte retrieval number. Model 2 employed multivariate logistic regression, and Model 3 applied inverse probability weighting (IPW) to assess fresh and cumulative live birth rates. For the IPW analysis, propensity scores for group assignment were estimated using multinomial logistic regression. Inverse probability weights were then calculated and applied to the outcome models. Covariate balance before and after weighting was assessed using standardized mean differences (SMDs), with values <0.1 considered acceptable. Details of weight construction and balance diagnostics for the IPW analysis of the primary outcome (CLBR) are shown in **Supplementary Table 4**. Results were presented as β coefficients or adjusted odds ratios (aORs) with 95% confidence intervals (CIs), as appropriate. Covariates were selected according to the outcome being analyzed and included male age, female age, body mass index (BMI), infertility type, infertility duration, infertility causes, COS protocols, fertilization method, embryonic period, embryo number, and hyperthyroidism duration. Detailed specifications are provided in the corresponding table notes.

All statistical analyses were conducted using IBM SPSS Statistics (version 22.0) and R (version 4.3.0). A 2-tailed P value <.05 was considered statistically significant.

## Results

### Patient and cycle characteristics

**Figure 1:**
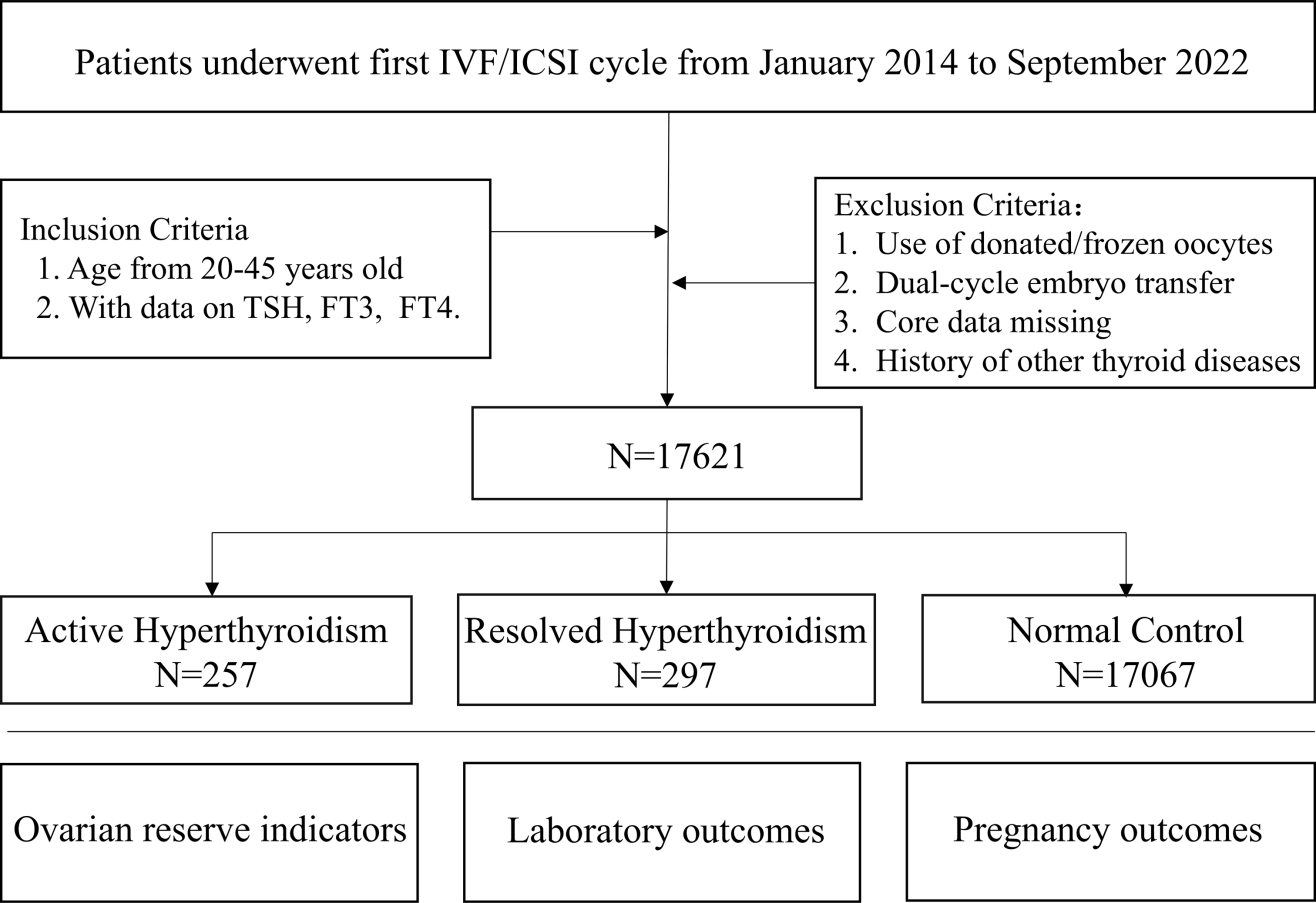
Flowchart. ^a^Embryo transfer involving embryos from two oocyte retrieval cycles. ^b^Other thyroid diseases include subclinical hypothyroidism, hypothyroidism, subclinical hyperthyroidism, thyroiditis, or thyroid cancer.

17,621 patients undergoing their first oocyte retrieval cycles were included. All patients had available TSH, FT3, and FT4 results within 3 months prior to oocyte retrieval. Patients were categorized into three groups: 257 patients in the AH group, 297 patients in the RH group, and 17067 in the NC group. **Figure 1** shows the flowchart and baseline characteristics are presented in **Table 1**. In **Table 1**, treatment-related categories represent current management in the AH group and prior treatment history in the RH group. All RH patients had been off treatment for at least 3 months before oocyte retrieval and remained off treatment throughout COS and ET.

**Table 1:** Patients’ general characteristics.

Variable	AH (1) N=257	RH (2) N=297	NC (3) N=17067	P1vs2	P1vs3	P2vs3	P all
Male Age (years)	37 (33-40)	37 (33-42)	36 (32-40)	0.395	0.024*	0.001*	<0.001*
Female Age (years)	34 (30-38)	33 (30-38)	32 (29-36)	0.400	<0.001*	<0.001*	<0.001*
BMI (kg/m^2^)	21.4 (19.7-23.2)	22.1 (20.1-23.7)	21.8 (19.9-24.0)	0.037*	0.035*	0.546	0.088
Infertility duration (years)	4.0 (2.0-6.0)	4.0 (2.4-6.0)	4.0 (2.0-6.0)	0.242	0.897	0.127	0.308
Infertility type, n (%)				0.306	0.774	0.239	0.477
Primary	128 (49.8)	135 (45.5)	8346 (48.9)				
Secondary	129 (50.2)	162 (54.5)	8721 (51.1)				
Infertility causes, n (%)				0.359	0.038*	<0.001*	<0.001*
Male factor	52 (20.2)	65 (21.9)	3230 (18.9)				
Tubal factor	90 (35.0)	88 (29.6)	7579 (44.4)				
Endometriosis	6 (2.3)	15 (5.1)	403 (2.4)				
Reduced ovarian reserve	3 (1.2)	5 (1.7)	172 (1.0)				
Mixed	106 (41.2)	124 (41.8)	5683 (33.3)				
Previous spontaneous abortion, n (%)	28 (11.3)	41 (14.2)	2131 (12.7)	0.376	0.579	0.485	0.588
Serum TSH (mIU/L)	0.002 (0.000-0.017)	1.274 (0.820-2.355)	1.524 (1.091-2.121)	<0.001*	<0.001*	0.002*	<0.001*
FT3 (pmol/L)	8.075 (5.908-13.063)	4.190 (3.820-4.500)	4.320 (4.010-4.660)	<0.001*	<0.001*	<0.001*	<0.001*
FT4 (pmol/L)	23.100 (19.415-27.435)	13.340 (12.455-14.525)	13.260 (12.400-14.180)	<0.001*	<0.001*	0.027*	<0.001*
Duration of hyperthyroidism (months)^a^	6 (3-15)	12 (6-28)	NA	<0.001*	NA	NA	NA
Treatment history/status^b^, n (%)				<0.001*	NA	NA	NA
Antithyroid Drugs	229 (90.5)	214 (75.1)	NA				
Observation only	19 (7.5)	13 (4.6)	NA				
I131 therapy	0	34 (11.9)	NA				
Surgery	5 (2.0)	24 (8.4)	NA				
Off treatment during COS and ET, n (%)	19 (7.5)	297 (100.0)	NA				
Basal FSH (IU/L)	5.8 (4.5-7.0)	5.9 (4.6-7.3)	5.4 (4.3-6.8)	0.499	0.039*	0.002*	0.001*
AFC	15 (10-20)	14 (9-20)	16 (11-23)	0.305	0.018*	<0.001*	<0.001*
AMH (ng/ml)	2.5 (1.1-5.0)	2.3 (1.0-4.0)	3.2 (1.7-5.7)	0.280	<0.001*	<0.001*	<0.001*

Continuous data are reported as medians (first quartile, third quartile) and analyzed by Mann-Whitney U tests.

Categorical data are reported as n (%) and analyzed by c^2^.

BMI, body mass index; TSH, thyroid-stimulating hormone; FT3, free triiodothyronine; FT4, free thyroxine; FSH, follicle stimulating hormone; AMH, anti-Müllerian hormone; AFC, antral follicle count.

^a^ The definition of hyperthyroid duration is the time from diagnosis to oocyte retrieval in AH patients and diagnosis to TH normalization in RH patients.

^b^ For the AH group, treatment categories indicate the management status at or immediately before oocyte retrieval. For the RH group, treatment categories indicate prior treatment history before resolution of hyperthyroidism. All patients in the RH group had discontinued antithyroid treatment for at least 3 months before oocyte retrieval and remained off treatment during controlled ovarian stimulation and embryo transfer.

*P<0.05.

Among the three groups, the median (IQR) serum TSH levels were 0.002 mIU/L (0.000-0.017), 1.274 mIU/L (0.820-2.355), and 1.524 mIU/L (1.091-2.121), respectively. The median (IQR) FT3 levels were 8.075 pmol/L (5.908-13.063), 4.190 pmol/L (3.820-4.500), and 4.320 pmol/L (4.010-4.660), respectively. The median (IQR) FT4 levels were 23.100 pmol/L (19.415-27.435), 13.340 pmol/L (12.455-14.525), and 13.260 pmol/L (12.400-14.180), respectively.

No statistically significant differences in baseline characteristics were observed between the AH group and the RH group, except for body mass index, which was higher in the RH group. Basal follicle-stimulating hormone (FSH), antral follicle count (AFC), and anti-Müllerian hormone (AMH) levels were comparable between the two groups. However, group AH demonstrated a higher mature rate and a greater number of viable embryos on day 3.

Compared with NC patients, AH and RH patients were older and had lower AMH, AFC levels, and higher FSH levels. The AH group also exhibited a higher oocyte retrieval cancellation rate, fewer retrieved oocytes, and fewer cleavage-stage embryos. Additionally, patients in the RH group were more likely to undergo ICSI and had a lower blastocyst formation rate (**Table 2**).

**Table 2:** *In vitro* fertilization treatments and laboratory outcomes.

Variable	AH (1) N=257	RH (2) N=297	NC (3) N=17067	P1vs2	P1vs3	P2vs3	P all
COS protocol				0.787	0.024*	0.007*	0.002*
GnRH-a	92 (35.8)	109 (36.7)	7500 (43.9)				
GnRH-ant	137 (53.3)	151 (50.8)	8144 (47.7)				
Others	28 (10.9)	37 (12.5)	1423 (8.3)				
Gn duration (days)	10 (8-12)	10 (8-12)	10 (9-12)	0.200	<0.001*	0.013*	<0.001*
Gn dosage (IU)	1650 (1200-2450)	1900 (1350-2700)	1775 (1275-2475)	0.008*	0.070	0.036*	0.021*
Follicles≥14mm on hCG day	4 (0-10)	3 (0-9)	6 (1-11)	0.217	0.005*	<0.001*	<0.001*
Peak E2 value (pg/ml)	9739 (4896-14767)	8289 (3670-13954)	9834 (5719-14700)	0.087	0.393	<0.001*	0.001*
P4 value on hCG day (ng/ml)	2.0 (1.3-3.1)	2.0 (1.3-3.3)	2.1 (1.4-3.1)	0.709	0.432	0.823	0.718
Endometrial thickness on hCG day (mm)	9.9 (8.0-11.7)	10.0 (7.6-11.5)	10.0 (8.5-11.8)	0.685	0.068	0.015*	0.010*
Oocyte retrieval canceled, n (%)	9 (3.5%)	9 (3.0%)	256 (1.5%)	0.917	0.009*	0.002*	0.004*
No. of oocytes retrieved	10 (5-15)	8 (4-14)	11 (7-17)	0.084	0.001*	<0.001*	<0.001*
Oocyte mature rate, n (%)	2292/2739 (83.7)	2292/2847 (80.5)	172477/209155 (82.5)	0.002*	0.096	0.006*	0.006*
Women obtain no viable oocyte, n (%)	12 (4.8)	6 (2.1)	245 (1.5)	0.127	<0.001*	0.530	<0.001*
Fertilization methods^a^, n (%)				0.428	0.174	0.004*	0.005*
IVF	170 (72.0)	193 (68.4)	12599 (76.1)				
ICSI	66 (28.0)	89 (31.6)	3967 (23.9)				
Cleavage embryo rate^b^, n (%)	1456/2292 (63.5)	1463/2292 (63.8)	112955/172477 (65.5)	0.830	0.049*	0.097	0.038*
No. of viable embryos on Day 3	3 (1-7)	3 (1-5)	4 (2-7)	0.003*	0.175	<0.001*	<0.001*
Blastocyst formation rate, n (%)	576/1218 (47.3)	522/1142 (45.7)	47143/95461 (49.4)	0.441	0.146	0.014*	0.017*
Total no. of viable embryos	3 (2-5)	2 (1-4)	4 (2-6)	0.014*	0.010*	<0.001*	<0.001*

Continuous data are reported as medians (first quartile, third quartile) and analyzed by Mann-Whitney U tests.

Categorical data are reported as n (%) and analyzed by c^2^.

COS, controlled ovarian stimulation; GnRH, gonadotropin-releasing hormone; Gn, gonadotropin; E2, estradiol; P4, progesterone; IVF, *in vitro* fertilization; ICSI, intracytoplasmic sperm injection.

^a^ Those who canceled oocyte retrieval or obtained no oocytes were excluded from the comparison between IVF and ICSI.

^b^ The cleavage rate was calculated by dividing the number of cleaved embryos by the number of mature oocytes.

*P<0.05.

The embryo transfer situations of the three groups were comparable, regardless of the embryo type or the number of embryos transferred, whether in fresh cycles or FET cycles. The detailed information can be found in **Supplementary Table 1**.

### Pregnancy outcomes

**Figure 2:**
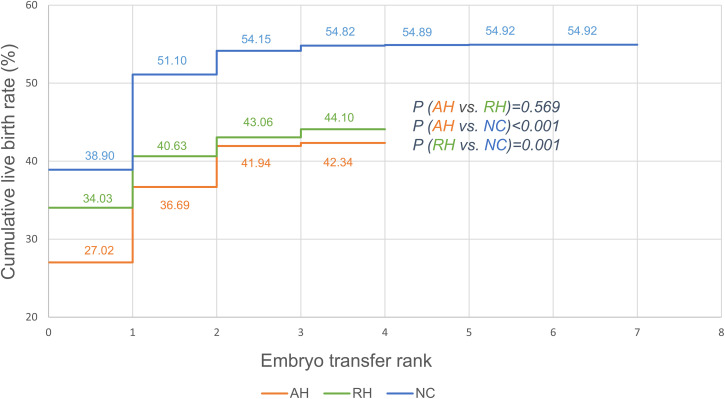
Kaplan–Meier curves of cumulative live birth rates for the three groups. The curves show the cumulative live birth rates (CLBRs) for three groups as the number of embryo transfer cycles increased. Compared to the control group, a significant decrease in the CLBR was observed in the Active Hyperthyroidism (AH) group and the Resolved Hyperthyroidism (RH) group (P < 0.001; P = 0.001), while no significant difference between group AH and group RH (P = 0.569).

**Table 3** and **Figure 2** summarize pregnancy outcomes across the three groups. Fresh embryo transfers were performed in 113 patients (45.6%) in the AH group, 121 patients (42.0%) in the RH group, and 8055 patients (47.9%) in the NC group. The implantation rate in the NC group (43.2%) was significantly higher than that in the AH group (31.0%) and the RH group (39.0%); however, the difference between the AH and RH groups was not statistically significant. Compared with the AH group, both the clinical pregnancy rate (CPR) and LBR were significantly higher in the NC group, while no significant differences were observed between the AH and RH groups.

**Table 3:** Cumulative outcomes after one entire ART cycle.

Variable	AH (1) (n=248)	RH (2) (n=288)	NC (3) (n=16811)	P1vs2	P1vs3	P2vs3	P all
Fresh ET cycles	113	121	8055				
No. of embryos transferred	171	182	12296				
Implantation rate, n (%)	53 (31.0)	71 (39.0)	5308 (43.2)	0.115	0.001*	0.261	0.003*
Biochemical pregnancy rate, n (%)	3 (2.7)	3 (2.5)	201 (2.5)	1.000	1.000	1.000	0.994
Clinical pregnancy rate, n (%)	44 (38.9)	60 (49.6)	4283 (53.2)	0.132	0.004*	0.489	0.008*
Early miscarriage rate, n (%)	8 (18.2)	8 (13.3)	524 (12.2)	0.498	0.232	0.797	0.476
LBR per ET	36 (31.9)	49 (40.5)	3517 (43.7)	0.216	0.016*	0.545	0.034*
FET cycles	211	202	14762				
No. of embryos transferred	313	301	21414				
Implantation rate, n (%)	113 (36.1)	121 (40.2)	8506 (39.7)	0.296	0.194	0.866	0.422
Biochemical pregnancy rate, n (%)	6 (2.8)	5 (2.5)	510 (3.5)	1.000	0.769	0.573	0.670
Clinical pregnancy rate, n (%)	94 (44.5)	102 (50.5)	7239 (49.0)	0.267	0.220	0.733	0.394
Early miscarriage rate, n (%)	16 (17.0)	15 (14.7)	1181 (16.3)	0.657	0.854	0.662	0.893
LBR per ET	69 (32.7)	78 (38.6)	5716 (38.7)	0.249	0.087	1.000	0.204
Cumulative outcomes							
CLBR, n (%)	105 (42.3)	127 (44.1)	9233 (54.9)	0.747	<0.001*	<0.001*	<0.001*
TTLB, days	296 (249-402)	288 (256-368)	288 (256-352)	0.988	0.759	0.755	0.910
Time from Gn to LB, days	307 (263-414)	302 (269-384)	301 (266-366)	0.857	0.875	0.667	0.901

Patients who cancelled oocyte retrieval were excluded from the outcome analysis. TTLB and time from Gn to LB were calculated among patients with live birth. Categorical data are reported as n (%) and analyzed by c^2^. ET, embryo transfer; LBR, live birth rate; FET, frozen embryo transfer; LB, live birth; CPR, cumulative pregnancy rate; CLBR, cumulative live birth rate; TTLB, time to live birth. Time from Gn to LB, time from gonadotropin start to live birth. *P<0.05.

211, 202, and 14,762 FET cycles with embryo transfer were conducted in the AH, RH, and NC groups, respectively. No significant differences were observed in implantation rate, biochemical pregnancy rate, CPR, LBR, or early abortion rate among the three groups.

Regarding cumulative outcomes, the CLBR was 42.3% (105/248) in the AH group, 44.1% (127/288) in the RH group, and 54.9% (9233/16,811) in the NC group (**Table 3**). Time from oocyte retrieval to live birth (TTLB) was comparable among the three groups (296 (249-402) vs. 288 (256-368) vs. 288 (256-352), P = 0.910). By the end of follow-up, ongoing pregnancies were reported in 3 patients (1.2%) in the AH group, 4 patients (1.4%) in the RH group, and 64 patients (0.4%) in the NC group. Absolute differences in major IVF outcomes between groups were shown in **Supplementary Table 5**.

### Perinatal outcomes

As shown in **Supplementary Table 2**, 82 AH patients and 101 RH patients had singleton live births. No significant differences were found in maternal complications and neonatal outcomes between the two groups.

### Adjusted models

Three statistical models were used, with results presented in **Table 4**. For the number of oocytes retrieved, a multiple linear regression model indicated no significant differences among the three groups.

**Table 4:** Unadjusted and adjusted effect estimates for clinical outcomes.

Variable	AH	RH	NC
No. of oocytes retrieved			
Unadjusted β^a^ (95%CI)	Ref	-1.159 (-2.517, 0.199)	1.397 (0.389, 2.405)*
Adjusted β, Model1^ad^ (95%CI)	Ref	-1.033 (-2.267, 0.202)	0.588 (-0.353, 1.529)
Live birth in fresh cycles			
Unadjusted OR^a^ (95%CI)	Ref	1.456 (0.853-2.501)	1.658 (1.122-2.495)*
Adjusted OR, Model 2^be^ (95%CI)	Ref	1.503 (0.866-2.625)	1.524 (1.019-2.318)*
Adjusted OR, Model 3^ce^(95%CI)	Ref	1.680 (0.972-2.924)	1.564 (1.045-2.379)*
Cumulative live birth			
Unadjusted ORa (95%CI)	Ref	1.074 (0.763-1.515)	1.659 (1.289-2.143)*
Adjusted OR, Model 2bf (95%CI)	Ref	1.174 (0.814-1.693)	1.451 (1.110-1.904)*
Adjusted OR, Model 3cf (95%CI)	Ref	1.339 (0.935-1.921)	1.549 (1.187-2.024)*

^a^ Effect estimates for oocyte count are presented as unstandardized β coefficients with 95% confidence intervals (CIs), based on linear regression models; for live birth outcomes, odds ratios (ORs) and 95% CIs, based on the univariate logistic regression analysis.

^b^ Adjusted odds ratios (aORs), 95% CIs, based on the multivariate binary logistic regression.

^c^ aORs and 95% CIs, based on the inverse probability weighting.

^d^ adjusted for female age, BMI, primary infertility, infertility durations, infertility causes, and COS protocols.

^e^ adjusted for male age, female age, BMI, primary infertility, infertility durations, infertility causes, COS protocols, fertilization methods, number of embryos transferred, cleavage embryo/blastocyst ET.

^f^ adjusted for male age, female age, BMI, primary infertility, infertility durations, infertility causes, COS protocols, and fertilization methods. ^*^P<0.05.

Compared with group AH, both the binary logistic regression and the IPW models indicated no significant difference in the LBR of fresh cycles and the CLBR in group RH. However, both models demonstrated an increased probability of LBR and CLBR in the NC group.

### Relationship between hyperthyroidism duration and live birth

Notably, the models demonstrated comparable oocyte retrieval and live birth rates between the AH and RH groups, suggesting that the status of hyperthyroidism may not significantly impact IVF outcomes. To further investigate the effect of hyperthyroidism, a subgroup analysis was performed among patients in the AH and RH groups. Of the 554 participants, specific medical histories were successfully obtained for 538 patients (97.1%). Among the 538 patients, 18 cycles were canceled; therefore, 520 were included in the analysis of association between hyperthyroidism duration and CLBR. The duration of hyperthyroidism was shorter in the AH group than in the RH group (**Table 1**).

**Figure 3:**
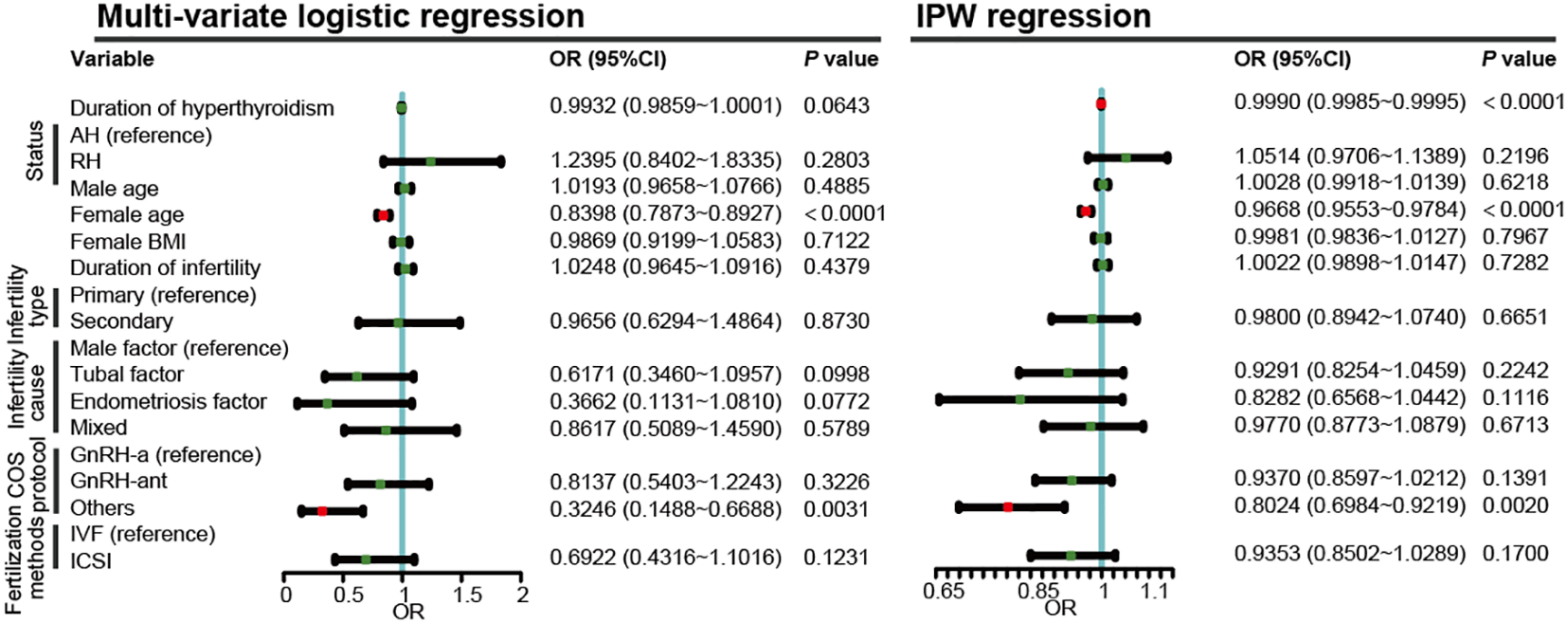
Association of hyperthyroidism duration and status with cumulative live birth among patients with hyperthyroidism history. IPW regression analyses demonstrated a significant adverse effect of hyperthyroidism duration on cumulative live birth rates (aOR=0.9990 (0.9985-0.9995), P<0.001), with no association observed for disease status (active vs. resolved, P>0.05).

The duration of hyperthyroidism was included in the analysis to evaluate its association with the number of oocytes, LBRs in fresh cycles, and CLBRs. As shown in **Supplementary Table 3**, the study revealed that the duration of hyperthyroidism is associated with CLBR. The binary logistic regression model suggested a trend of negative correlation between the duration and the outcome (P = 0.064). When considering the IPW model, this negative correlation was more completely exposed, with aOR=0.9990 (0.9985-0.9995), P < 0.001. Specifically, longer durations of hyperthyroidism were associated with a lower probability of achieving a live birth within the oocyte retrieval cycle. To visually illustrate the influence of each variable in cumulative live birth, a forest plot of regression coefficients is presented in **Figure 3**.

## Discussion

In this retrospective study, 17,621 patients were included to investigate the association between hyperthyroidism status and IVF outcomes. The results suggested that preconception hyperthyroidism was associated with poorer IVF outcomes. Notably, the IVF and perinatal outcomes in patients with resolved hyperthyroidism were not clearly better than those in patients with active hyperthyroidism. Subgroup analysis indicated a longer duration of hyperthyroidism was associated with a lower likelihood of live birth, suggesting the adverse effect of hyperthyroidism on IVF outcomes may persist.

In the comparison between the AH and RH groups, we observed a remarkable degree of similarity in baseline clinical characteristics, interventions, and pregnancy outcomes, even after adjusting for relevant variables. Notably, the two groups had comparable AMH, FSH, and AFC levels, suggesting similar ovarian reserves. In the context of fresh embryo transfers, patients in the RH group had marginally higher embryo implantation rates and clinical pregnancy rates compared with those in the AH group (39.0% vs 31.0% and 49.6% vs 38.9%, respectively), which might reflect the impact of hyperthyroidism on the endometrial environment. However, these differences were not statistically significant. When examining cumulative outcomes, no significant differences were noted between the two groups. Using NC group as the reference, the AH group showed poorer reproductive outcomes. In contrast, the RH group generally did not demonstrate a statistically significant improvement over AH.

To our knowledge, this is the first study to investigate IVF outcomes in patients with resolved hyperthyroidism. A 2019 study reported that women with hyperthyroidism had a reduced likelihood of live birth following assisted reproductive technology (ART) treatments compared with women without thyroid disease (6). This finding was corroborated by the comparison between the AH group and NC group in our study. In this study, we conducted a comprehensive assessment of reproductive potential and outcomes, including metrics such as CLBR and time to live birth, which provided a more robust and accurate measure of treatment efficacy and safety. The use of CLBR as an evaluation metric is particularly meaningful for infertile couples and clinicians (17). Importantly, our study included patients with normalized thyroid function but a history of hyperthyroidism. Results from these patients indicate that normalization of thyroid function may not fully reverse the adverse reproductive effects associated with prior hyperthyroid exposure. Further analysis of 520 evaluable patients clarified the duration of hyperthyroid exposure, as shown in **Figure 3**. Our findings suggest that the duration of hyperthyroid exposure, rather than remission status, is more closely associated with poorer reproductive outcomes. This suggests that hyperthyroidism-induced ovarian damage may reflect a cumulative effect. The longer the exposure, the greater the damage.

Previous research has indicated that T3 and T4 are present in human follicular fluid and involved in hCG-induced cyclic adenosine monophosphate (cAMP) responses (18, 19). These hormones also play a role in regulating nitric oxide synthase activity and participate in signaling pathways crucial for follicular development (20, 21). TH receptors (THRs) are expressed in various ovarian microenvironment cells, including granulosa and stromal cells, and are critical for folliculogenesis, steroidogenesis, and follicular fluid synthesis (22, 23). Exposure to elevated TH levels has been shown to increase the expression of inflammation-related genes in ovarian surface epithelial cells and activate ERK1/2 signaling in granulosa cells, potentially reshaping the ovarian microenvironment and impairing ovarian reserve (24–26). Further research is required to elucidate the mechanisms underlying these effects.

The effects of THs on the endometrium have been extensively studied. Aghajanova et al. reported that THRs and TSH receptors are widely expressed in the endometrium, with expression levels varying throughout the menstrual cycle (27, 28). Inuwa and Williams observed that THs may interfere with estrogen activity in target tissues, including the reproductive tract (29). Additionally, in hyperthyroid states, sex hormone-binding globulin (SHBG) levels are elevated, leading to increased circulating total estradiol levels, normal or reduced free estradiol levels, and decreased estradiol metabolic clearance rates (30). Through these mechanisms, THs may influence endometrial proliferation and maturation by regulating the hypothalamic-pituitary-ovarian axis, thereby impacting embryo implantation and early development (31, 32). However, the cyclical regeneration of the endometrium raises questions about whether pre-pregnancy TH effects on the endometrium persist into the embryo transfer stage (33). A study by Karmon et al. demonstrated that donor TSH levels were associated with clinical pregnancy outcomes in oocyte donation cycles, while recipient TSH levels were not associated with IVF outcomes (34). Although this study focused on patients with normal or slightly elevated TSH levels, it highlighted that the critical impact of THs on clinical pregnancy outcomes is primarily related to their effects on oocytes before embryo implantation rather than on the endometrium.

2017 ATA and 2021 ETA guidelines recommend achieving stable euthyroidism before pregnancy, especially before IVF (15, 16). However, the available evidence remains limited. Therefore, our findings are not in conflict with current guidelines, but may provide additional clinical evidence in this area. More specifically, our findings also suggest that the impact of prior overt hyperthyroidism on reproductive outcomes may not completely disappear even after thyroid function normalizes. In this study, the CLBR in the RH group was not clearly higher than that in the AH group, and longer disease duration was associated with a lower CLBR. This suggests that while achieving thyroid function control is important, repeatedly delaying reproductive plans while awaiting long-term disease resolution may not necessarily improve IVF outcomes. Therefore, for women with hyperthyroidism who desire pregnancy, timely endocrine treatment should be accompanied by early fertility counseling and individualized reproductive planning.

For future research, it would be worthwhile to assess further the impact of treatment methods and age of onset. Future studies will explore the mechanism of hyperthyroidism’s effect on the ovaries. Therefore, we can establish an optimal strategy for women with different disease course conditions.

This study has several limitations. First, the retrospective nature inherently led to imbalances between groups. The history of hyperthyroidism obtained through telephone interviews may be affected by recall bias. Second, thyroid antibody testing was not routinely available in our pre-IVF assessment, and only a subset of patients had antibody results; therefore, these data were not included in analysis. As a result, the etiology of hyperthyroidism could not be reliably classified for the entire cohort. Third, the RH group includes patients with euthyroidism via antithyroid drugs, surgery, or I131; small subgroup sizes limited analysis of treatment-related IVF outcomes. In addition, TTLB was calculated from oocyte retrieval and cannot directly answer whether IVF should be delayed until euthyroidism. Finally, as this was a single-center study conducted in China, the results may not be generalizable to other populations or regions. To address these gaps, future prospective studies will include a systematic assessment of thyroid antibody profiles alongside continuous thyroid function monitoring.

In total, we comprehensively investigated the impact of hyperthyroidism on clinical outcomes in the IVF population. Our findings indicate that the clinical outcomes in patients with resolved hyperthyroidism were not superior to those with active hyperthyroidism. Additionally, the duration of hyperthyroidism was negatively correlated with IVF outcomes. Although restoration of euthyroidism before IVF remains clinically necessary, our findings suggest that prior hyperthyroid exposure may still be associated with adverse reproductive effects. These results highlight the importance of timely endocrine management and early reproductive counseling in women with hyperthyroidism who desire pregnancy.

## Data Availability

Due to patient privacy, de-identified raw data will be made available upon reasonable request.
