# Supplementary material for: Resolved hyperthyroidism before IVF is not associated with improved cumulative live birth rates: a retrospective cohort study
Source: Front Endocrinol (Lausanne). 2026 Jun 2;17:1749355. doi: 10.3389/fendo.2026.1749355 (PMC13273757; doi:10.3389/fendo.2026.1749355)
Supplement: Supplementary file 1 [file Table1.docx]

**Supplements Materials**

Supplemental Table 1. Specific embryo-transfer information.

Supplemental Table 2. Perinatal outcomes of singleton live births in patients with a diagnosis of hyperthyroidism.

Supplemental Table 3. Association between clinical outcomes and hyperthyroidism duration in diagnosed patients.

Supplemental Table 4. Construction and balance diagnostics of inverse probability weighting (IPW) for cumulative live birth rate.

Part A. Weight construction. Part B. Distribution of weights. Part C.Covariate balance before and after weighting.

Supplemental Table 5. Absolute differences in major IVF outcomes between groups.

**Supplemental Table 1. Specific embryo-transfer information.**

|  | **AH (1)**  **(n=248)** | **RH (2)**  **(n=288)** | **NC (3)**  **(n=16811)** | **P1vs2** | **P1vs3** | **P2vs3** | **P all** |
| --- | --- | --- | --- | --- | --- | --- | --- |
| **Fresh ET cycles** | 113 (45.6) | 121 (42.0) | 8055 (47.9) | 0.409 | 0.462 | 0.047* | 0.108 |
| No. of embryos transferred | 171 | 182 | 12296 |  |  |  |  |
| embryos transferred per ET, n(%) |  |  |  | 0.889 | 0.780 | 0.625 | 0.855 |
| 1 | 55 (48.7) | 60 (49.6) | 3814 (47.3) |  |  |  |  |
| 2 | 58 (51.3) | 61 (50.4) | 4241 (52.7) |  |  |  |  |
| Type of embryo transferred |  |  |  | 0.679 | 0.288 | 0.637 | 0.512 |
| Cleavage embryo | 92 (81.4) | 101 (83.5) | 6848 (85.0) |  |  |  |  |
| Blastocyst | 21 (18.6) | 20 (16.5) | 1207 (15.0) |  |  |  |  |
| **FET cycles** |  |  |  |  |  |  |  |
| No. of FET with transfer | 211 | 202 | 14762 |  |  |  |  |
| No. of embryos transferred | 313 | 301 | 21414 |  |  |  |  |
| embryos transferred per ET, n(%) |  |  |  | 0.892 | 0.342 | 0.263 | 0.345 |
| 1 | 109 (51.7) | 103 (51.0) | 8110 (54.9) |  |  |  |  |
| 2 | 102 (48.3) | 99 (49.0) | 6652 (45.1) |  |  |  |  |
| Type of embryo transferred |  |  |  | 0.845 | 0.097 | 0.179 | 0.106 |
| Cleavage embryo | 74 (35.1) | 69 (34.2) | 4400 (29.8) |  |  |  |  |
| Blastocyst | 137 (64.9) | 133 (65.8) | 10362 (70.2) |  |  |  |  |

Note: Categorical data are reported as n (%) and analyzed by c^2^. FET=frozen embryo transfer. *P<0.05

**Supplemental Table 2. Perinatal outcomes of singleton live births in patients with a diagnosis of hyperthyroidism.**

|  | **AH**  **(n=82)** | **RH(n=101)** | **P value** |
| --- | --- | --- | --- |
| Maternal complications^a^ , n (%) | 6 (7.3) | 7 (6.9) | 0.919 |
| Preterm delivery, <37 wk, n (%) | 8 (9.8) | 7 (6.9) | 0.488 |
| Cesarean delivery, n (%) | 41 (50.0) | 51 (50.5) | 0.947 |
| Male gender, n (%) | 41 (50.0) | 52 (51.5) | 0.842 |
| Low birth weight, <2,500 g, n (%) | 3 (3.7) | 7 (6.9) | 0.516 |
| Birth defect ^b^, n (%) | 2 (2.4) | 3 (3.0) | 1.000 |
| Thyroid dysfunction in infants, n (%) | 2 (2.4) | 1 (1.0) | 0.588 |

Notes: ^a^ Maternal complications included hypertensive disorders of pregnancy (HDP), gestational diabetes, placenta previa, and thyroid crisis.

^b^ Birth defects included structural abnormalities (such as cleft lip and palate, atrial septal defects) and functional impairments (such as hearing loss).

*P<0.05

**Supplemental Table 3. Association between clinical outcomes and hyperthyroidism duration in diagnosed patients.**

|  | **Duration** | **P value** |
| --- | --- | --- |
| **No. of oocytes** |  |  |
| Unadjusted β^a^ (95%CI) | -0.009 (-0.031 to 0.013) | 0.414 |
| Adjustedβ, Model 1^bd^ (95%CI) | 0.003 (-0.017 to 0.023) | 0.782 |
| **Live birth in fresh cycles** |  |  |
| Unadjusted OR^a^ (95%CI) | 0.994 (0.983-1.002) | 0.196 |
| Adjusted OR, Model 2^be^ (95%CI) | 0.994 (0.982-1.004) | 0.269 |
| Adjusted OR, Model 3^be^ (95%CI) | 0.999 (0.997-1.001) | 0.283 |
| **Cumulative live birth** |  |  |
| Unadjusted OR^a^ (95%CI) | 0.9911 (0.9845-0.9977) | 0.008* |
| Adjusted OR, Model 2^bf^ (95%CI) | 0.9932 (0.9859-1.0001) | 0.064 |
| Adjusted OR, Model 3^cf^ (95%CI) | 0.9990 (0.9985-0.9995) | <0.001* |

Note: Duration was analyzed as a continuous variable in months. 520 patients with available disease duration and non-canceled retrieval cycle were included. For the association between hyperthyroidism and cumulative live birth, effect estimates are presented to four decimal places because the effect size per 1-month increase was very small; using three decimal places could mask the direction of the association due to rounding.

^a^ Effect estimates for oocyte count are presented as unstandardized β coefficients (per 1-month increase in duration) with 95% confidence intervals (CIs), based on linear regression models; for live birth outcomes, odds ratios (ORs) and 95% CIs, based on the univariate logistic regression analysis.

^b^ Adjusted odds ratios (aORs), 95% CIs, based on the multivariate binary logistic regression.

^c^ aORs and 95% CIs, based on the inverse probability weighting.

^d^ adjusted for hyperthyroidism status, female age, BMI, primary infertility, infertility durations, infertility causes, and COS protocols.

^e^ adjusted for hyperthyroidism status, male age, female age, BMI, primary infertility, infertility durations, infertility causes, COS protocols, fertilization methods, number of embryos transferred, cleavage embryo/ blastocyst ET.

^f^ adjusted for hyperthyroidism status, male age, female age, BMI, primary infertility, infertility durations, infertility causes, COS protocols, and fertilization methods.^*^P<0.05

**Supplemental Table 4.** **Construction and balance diagnostics of inverse probability weighting (IPW) for cumulative live birth rate.**

**Part A. Weight construction.**

| **Item** | **Description** |
| --- | --- |
| **Exposure groups** | AH, RH, and NC |
| **Propensity score model** | Multinomial logistic regression |
| **Estimand** | Average treatment effect for pairwise comparisons |
| **Weight type** | Unstabilized |
| **Weight truncation** | None |
| **Variables included in the propensity score model** | Male age, female age, BMI, primary infertility, infertility durations, infertility causes, COS protocols, and fertilization methods. |
| **Software** | R version 4.3.0 with the ipw package (version 1.2) |

**Part B. Distribution of weights.**

| **Group** | **Mean** | **SD** | **Median** | **Minimum** | **Maximum** | **P1** | **P99** |
| --- | --- | --- | --- | --- | --- | --- | --- |
| **AH** | 1.0062 | 0.4489 | 0.9052 | 0.3136 | 3.0924 | 0.3921 | 2.5008 |
| **RH** | 1.0142 | 0.5066 | 0.8800 | 0.2992 | 2.6138 | 0.3362 | 2.4229 |
| **NC** | 1.0000 | 0.0137 | 0.9973 | 0.9755 | 1.1176 | 0.9802 | 1.0436 |

**Part C.Covariate balance before and after weighting.**

| **Variable** | **Before weighting SMD** | **After weighting SMD** |
| --- | --- | --- |
| **Male age** | 0.2068 | 0.0401 |
| **Female age** | 0.3031 | 0.0335 |
| **BMI** | 0.1910 | 0.0755 |
| **Primary infertility** | 0.0754 | 0.0312 |
| **Infertility duration** | 0.0726 | 0.0870 |
| **Infertility causes** |  |  |
| Male factor | 0.0794 | 0.0154 |
| Tubal factor | 0.3062 | 0.0273 |
| Endometriosis | 0.1595 | 0.0066 |
| Reduced ovarian reserve | 0.0710 | 0.0120 |
| Mixed | 0.1629 | 0.0433 |
| **COS protocols** |  |  |
| GnRH-a | 0.1636 | 0.0565 |
| GnRH-ant | 0.1089 | 0.0483 |
| Others | 0.1281 | 0.0113 |
| **Fertilization method** | 0.1579 | 0.0139 |

**Notes: Covariate balance before and after weighting was assessed by cobalt using standardized mean differences (SMDs), with values <0.1 considered acceptable.**

**Supplemental Table 5. Absolute differences in major IVF outcomes between groups.**

|  |  |  |  | absolute difference (%), (95% CI) | | |
| --- | --- | --- | --- | --- | --- | --- |
| Outcome | **AH,**  **n/N (%)** | **RH,**  **n/N (%)** | **NC,**  **n/N (%)** | **AH vs NC** | **RH vs NC** | **RH vs AH** |
| Clinical pregnancy rate in fresh cycles | 44/113 (38.9) | 60/121 (49.6) | 4283/8055 (53.2) | -14.2 (-23.3 to -5.2) | -3.6 (-12.6 to 5.4) | 10.7 (-2.0 to 23.3) |
| Live birth rate in fresh cycles | 36/113 (31.9) | 49/121 (40.5) | 3517/8055 (43.7) | -11.8 (-20.5 to -3.2) | -3.2 (-12.0 to 5.7) | 8.6 (-3.6 to 20.9) |
| Cumulative live birth rate | 105/248 (42.3) | 127/288 (44.1) | 9233/16811 (54.9) | -12.6 (-18.8 to -6.4) | -10.8 (-16.6 to -5.0) | 1.8 (-6.7 to 10.2) |

**Notes:Absolute differences are expressed as percentage-point differences in event rates between groups, with 95% confidence intervals (CIs). Negative values indicate a lower event rate in the first group than in the comparison group.**
